# Knockdown of miR-155 alleviates skin damage in rats with chronic spontaneous urticaria by modulating the JAK/STAT signaling pathway

**DOI:** 10.1186/s13223-024-00902-x

**Published:** 2024-06-29

**Authors:** Yue-peng An, Rui Yuan, Shan-shan Wang, Su-qing Yang, Qing Zhang

**Affiliations:** Department of Dermatology, The First Affiliated Hospital of Heilongjiang University of Chinese Medicine, No. 26 Heping Road, Xiangfang District, Harbin, 150040 Heilongjiang China

**Keywords:** miR-155, JAK/STAT signaling pathway, Chronic urticaria, Autoimmunity

## Abstract

**Objective:**

The aim of this study was to investigate the role and mechanisms of miR-155 in chronic spontaneous urticaria (CSU).

**Methods:**

The expression level of miR-155 in the skin tissues of patients with CSU and experimental rats were detected by RT-qPCR, followed by the measurement of the histamine release rate in the serum through the histamine release test. Besides, hematoxylin & eosin staining was used to observe the pathological changes of the skin tissues; Corresponding detection kits and flow cytometry to measure the changes of immunoglobulins, inflammatory cytokines and T cell subsets in the serum of rats in each group; and western blot to check the expression level of proteins related to JAK/STAT signaling pathway in the skin tissues.

**Results:**

Knockdown of miR-155 reduced the number and duration of pruritus, alleviated the skin damage, and decreased the number of eosinophils in CSU rats. Moreover, knockdown of miR-155 elevated the serum levels of IgG and IgM, decreased the levels of IgA and inflammatory cytokines, and reduced the proportion of CD4 + and CD4 + CD25 + T cells, as well as the CD4+/CD8 + ratio in CSU rats. However, Tyr705 intervention could reverse the effects of knockdown of miR-155 on CSU model rats. Furthermore, we found that knockdown of miR-155 significantly reduced the protein expression of IRF-9, as well as the P-JAK2/JAK2 and P-STAT3/STAT3 ratios in the skin tissues of CSU rats.

**Conclusion:**

Knockdown of miR-155 can alleviate skin damage and inflammatory responses and relieve autoimmunity in CSU rats by inhibiting the JAK/STAT3 signaling pathway.

## Introduction

Chronic urticaria (CU) is an immune-mediated inflammatory disease defined as persistent or intermittent urticaria for more than 6 weeks, with an incidence of 0.5–5% [1]. The clinical manifestations of CU are recurrent pruritus, wheals, angioedema, and inflammatory cell infiltration. Despite not life-threatening, CU seriously affects patients’ daily life and work [2]. CU includes chronic idiopathic urticaria and chronic spontaneous urticaria (CSU), among which CSU accounts for about 80% of CU cases, where the wheals and/or angioedema occur without an apparent external trigger [3]. CSU is considered when symptoms (wheals and/or angioedema) persist or recur for more than six weeks without a specific trigger and physical factors (such as pressure, cold, or heat), medications, or food additives do not explain the urticaria [4]. In addition to complicated pathogenesis and causes, CSU is also characterized with a high recurrence rate. Currently, antihistamines and glucocorticoids are the main therapeutic drugs for CSU; although both are effective in managing symptoms for many patients, their efficacy varies without a cure effect [5, 6]. Additionally, treatments such as Omalizumab, an anti-IgE monoclonal antibody, have shown significant efficacy, especially in cases resistant to traditional therapies [7]. However, the variability in patient response to these treatments highlights the complexity of CSU management and underscores the urgent need for new and effective approaches to treat CSU.

MicroRNAs (miRNAs) are endogenous non-coding RNAs containing 22 nt, which regulate protein synthesis and participate in a variety of cellular life activities by binding to the 3’ untranslated region of target messenger RNA. Some miRNAs are also associated with the progression of CSU. For instance, miR-125a-5p, highly expressed in the serum of patients with CSU, is considered as a potential biomarker of CSU [8]. Additionally, miR-155 stands out for its critical role in regulating immune and inflammatory responses. Prior studies have established miR-155 as a pivotal player in hematopoiesis, inflammation, and immunity, making it a molecule of interest in autoimmune and inflammatory disorders [9–11]. Research has shown elevated levels of miR-155 in several autoimmune conditions, where it contributes to the dysregulation of immune responses. Also, miR-155 modulates the expression of various genes involved in the immune response, influencing the development and function of immune cells such as T cells, B cells, and dendritic cells [12, 13]. Given the inflammatory nature of CSU and its characteristics such as recurrent pruritus, wheals, and angioedema, miR-155’s role in inflammation and immune responses suggests its potential involvement in CSU pathogenesis. However, it is unclear whether miR155 is involved in the progression of CSU.

The Janus kinase (JAK)/signal transducer and activator of transcription (STAT) pathway transmits information received from extracellular polypeptide signals directly to target gene promoters in the nucleus via transmembrane receptors, providing a mechanism for transcriptional regulation without second messengers [14]. JAK, required for many inflammatory cytokine signaling pathways, is implicated in the pathogenesis of chronic dermatitis, atopic dermatitis, and CSU [15–17]. It has been reported that JAK/STAT signaling is activated in skin tissues of patients with CSU, and a variety of related inflammatory factors and STAT proteins are involved in the occurrence and development of CSU [16]. This study was designed to explore the role and mechanism of miR-155 in CSU, as well as the effect of JAK/STAT signaling involved in it. In this study, the expression changes of miR-155 in skin tissues of patients with CSU and experimental rats were examined, and the role and related mechanisms of miR-155 in CSU were also explored by knocking down miR-155. This study suggested that miR-155 could be a potential therapeutic target for CSU.

## Materials and methods

### Clinical data

The skin and serum were collected from 20 patients with CSU (CSU group) diagnosed and treated in our hospital. Besides, normal skin and serum collected from 20 plastic surgery patients were set as controls (Normal group). The inclusion criteria of patients in CSU group were shown as follows: (1) age ≥ 18 years but ≤ 75 years; (2) the patients were in the active stage of the disease without antihistamine treatment; (3) the course of the disease was more than 8 weeks; (4) the patients consented to participate in this study. The inclusion criteria of patients in normal group were listed as follows: (1) without skin diseases and age paralleled with the CSU group; (2) undergoing plastic surgery in our hospital; (3) willing to participate in this study. The exclusion criteria applied to both the two groups were as follows: (1) with disease of vital organs such as lungs, kidneys, cardiovascular and cerebrovascular diseases; (2) with disease of the immune system; (3) lactating women or pregnant women; (4) treated with corticosteroids and immunosuppressants, and diagnosed with chronic inducible urticaria or urticarial vasculitis; (5) suffering from active atopic diseases, such as allergic rhinitis, atopic dermatitis or asthma. All procedures followed the ethical principles of medical research and all patients signed an informed consent. This study was approved by the Ethics Committee of The First Affiliated Hospital of Heilongjiang University of Chinese Medicine (HZYLLKY202009027).

### Construction and grouping of CSU animal models

Thirty-six healthy specific pathogen free (SPF) Sprague Dawley rats (6–8 weeks old, 180–200 g) were purchased from Hunan SJA Laboratory Animal Co.,Ltd. All animal experiments were approved by the Ethics Committee of The First Affiliated Hospital of Heilongjiang University of Chinese Medicine (HZYDWLLKY202003417) and performed in accordance with ethical guidelines. The rats were housed in an SPF environment with a stable temperature and humidity within their living environment as well as a consistent circadian rhythm of 12-hour light/dark cycle. After one week of adaptive feeding, the rats were subjected to the experiments. Furthermore, rats had ad libitum access to food and water.

The CSU rat model was constructed according to the previous method [17]. Aluminum hydroxide (Yuanye Bio-Technology, China) was dissolved in saline to make a solution with a final concentration of 10 mg/ml. Ovalbumin (1 mg, Sigma-Aldrich, St Louis, MO, USA) was added to aluminum hydroxide solution (1 ml) to prepare a suspension. Rats were intraperitoneally injected with 1 ml of suspension containing ovalbumin (1 mg). After 5 days, 1 ml of suspension containing ovalbumin (1 mg) and aluminum hydroxide was injected again. The number and maximum duration of pruritus in rats were observed each day, when they were significantly higher than those of normal control rats, it indicated that the CSU model was constructed successfully.

All animals were divided into 6 groups (*n* = 6 rats/group): (1) normal group: rats were intraperitoneally injected with the same amount of saline, and then intraperitoneally injected again with the same amount of saline after 5 days; (2) CSU group: no other treatment was given to rats after successful CSU modeling; (3) NC group: rats were injected with negative inhibitor after successful CSU modeling; (4) in-miR-155 group: CSU rats were injected with miR-155 inhibitor; (5) Tyr705 group: CSU rats were injected with JAK/STAT signaling pathway agonist Tyr705 (Absin, China) [4]; (6) in-miR-155 + Tyr705 group: CSU rats were injected with miR-155 inhibitor and Tyr705. Negative inhibitor and miR-155 inhibitor were both designed and constructed by BGI Genomics. Thirty min after the injection, the number and maximum duration of pruritus within one day were measured. The behaviors of the rats were defined as pruritus, such as rubbing their paws against the skin of their back, nose, or ears and then placing the paws back on the floor [18]. Upon completion of the measurements, the rats were euthanized by intraperitoneal injection of pentobarbital sodium (100 mg/kg), followed by collection of blood and back skin tissues with wheal or rash.

### RT-qPCR

Total RNA was extracted from skin tissues of patients and rats using miRNeasy Mini Kit (217,004, Qiagen, Düsseldorf, Germany). The concentration and purity of RNA were detected by NanoDrop. RNA was reversely transcribed to cDNA in accordance with the instructions of the Reverse Transcription Kit (TaKaRa, Japan). The target gene was synthesized using the Thermal Cycler Dice® Real Time System according to the instructions of the SYBR GREEN Kit (TaKaRa, Japan). U6 was used as an internal control. The relative expression of the target gene was calculated by the 2^-ΔΔCt^ method. The primer sequences were shown in Table 1.

**Table 1:** Primer Sequences for RT-qPCR

Gene Name	Primer sequences (5’ to 3’)
miR-155	F: CTGTAT-CAAAAGGCCAACTGAA
	R: GTGTCTATCCT-TATGAATCGCCA
U6	F: CTCGCTTCGGCAGCACATA
	R: AACGATTCACGAATTTGCGT

### Histamine release assay

Histamine release rate was determined according to the previous literature [4]. Serum from patients and rats was mixed with mast cells after incubation at 37 °C for 20 min. After centrifugation (3000 rpm, 15 min), the supernatant was collected. The histamine content in the supernatant was detected by Histamine ELISA Kit (COIBO, Shanghai, China), and the histamine release rate was calculated.

### Hematoxylin and eosin (H&E) staining

After the rats were euthanized, their skin tissues were isolated and washed with saline (4 °C) to remove surface bloodstains. Next, the tissues were fixed with 4% paraformaldehyde for 24 h, embedded in paraffin and then cut into 5 μm serial sections. Later, the tissue sections were deparaffinized with xylene, followed by a gradient ethanol to remove xylene. Subsequently, the sections were stained with hematoxylin (Solarbio, China) for 10 min, differentiated in 10% hydrochloric acid in ethanol for 10 s, and immersed in 1% ammonia for 30 s. The sections were subsequently stained with eosin (Solarbio, China) for 3 min. Finally, the tissue morphology was observed under a light microscope (Olympus, Tokyo, Japan), the photographs were collected, and eosinophil counts were performed.

### Determination of immunoglobulins

The levels of immunoglobulin G (IgG), immunoglobulin M (IgM) and immunoglobulin A (IgA) in rat serum were detected by immunoturbidimetry according to the instructions of the immunoglobulin detection kits (Jining Shiye, Shanghai, China).

### Detection of inflammatory cytokines and immunoglobulin E

Rat serum was collected, then the levels of interleukin (IL)-18, IL-6, IL-2, interferon-gamma (IFN-γ) and immunoglobulin E (IgE) in rat serum were detected based on the instructions of ELISA kit (Keshun, Shanghai, China). The samples and antibodies were sequentially added into the ELISA plate and incubated at room temperature for 1 h. Then, the diluted horseradish peroxidase-labeled conjugated antibody was added into each well for another incubation at room temperature for 0.5 h. After that, the supernatant was discarded, and 100 µL of colorimetric substrate 3’-3-5’-5-tetramethylbenzidine (TMB) was added into each well and incubated in a dark box at room temperature for 30 min. Next, 100 µL of stop solution was added to each well, and the absorbance value at 450 nm was measured by a Microplate Reader (Decca, China). Finally, the corresponding cytokine levels in each sample were calculated according to the standard curve.

### Flow cytometry

The peripheral blood ( 100 µL) of rats was gathered, supplemented with 10 µL of antibody, and then incubated for 20 min at room temperature away from light. Later, erythrocytes were lysed with Red Blood Cell Lysis Buffer (Thermo Fisher, USA) for 10 min, centrifuged for 5 min, washed with 2 ml of phosphate-buffered saline and then resuspended. Lastly, flow cytometry (BD FACSCanto II, BD Biosciences, Franklin lakes, NJ, USA) was used to detect the proportion of CD3+, CD4+, CD8 + T, CD4 + CD25 + Treg.

### Western blot

Total proteins were extracted from rat skin tissues on ice using RIPA Lysis Buffer. Then, the protein concentration was determined by a BCA kit (Beyotime, China). Next, the total protein was added to 5 × sodium dodecyl sulfate-polyacrylamide gel electropheresis (SDS-PAGE) Protein Sample Buffer (Solarbio, China) and boiled for 5 min. Protein samples were separated by SDS-PAGE and transferred to polyvinylidene difluoride (PVDF) membranes (Rio-Bad, USA), followed by blocking with 5% skimmed milk powder for 2 h. Subsequently, the PVDF membranes were incubated with primary antibodies at 4 °C overnight. Primary antibodies were purchased from Abcam (Cambridge, MA, USA), including JAK2 (ab108596, 1 : 5000), p-JAK2 (ab32101, 1 : 10,000), STAT3 (ab68153, 1 : 2000), p-STAT3 (ab267373, 1 : 1000), IRF-9 ( ab271043, 1 : 1000), and GAPDH (ab8245, 1 : 10,000). The PVDF membranes were rinsed with Tris-buffered saline containing Tween 20 (TBST, Solarbio, China) and incubated with secondary antibody (Abcam, USA) at room temperature for 2 h. Next, BCL luminescent solution (Solarbio, China) was uniformly added to the membranes, which were subsequently scanned and photographed with FluorchemHD2 imaging system. Gray scale values of protein bands were analyzed by Image J. GAPDH was used as an internal reference protein to calculate the relative expression of the target proteins.

### Statistical analysis

The experimental data were analyzed statistically by SPSS 24.0 software. The differences between two groups were analyzed by t test, and the differences among multiple groups were compared by one-way analysis of variance. The results were expressed as mean ± standard deviation (SD), and *P* < 0.05 was used as the criterion for a significant difference.

## Results

### Highly expressed miR-155 in skin tissues of patients with CSU and rat models

**Fig. 1:**
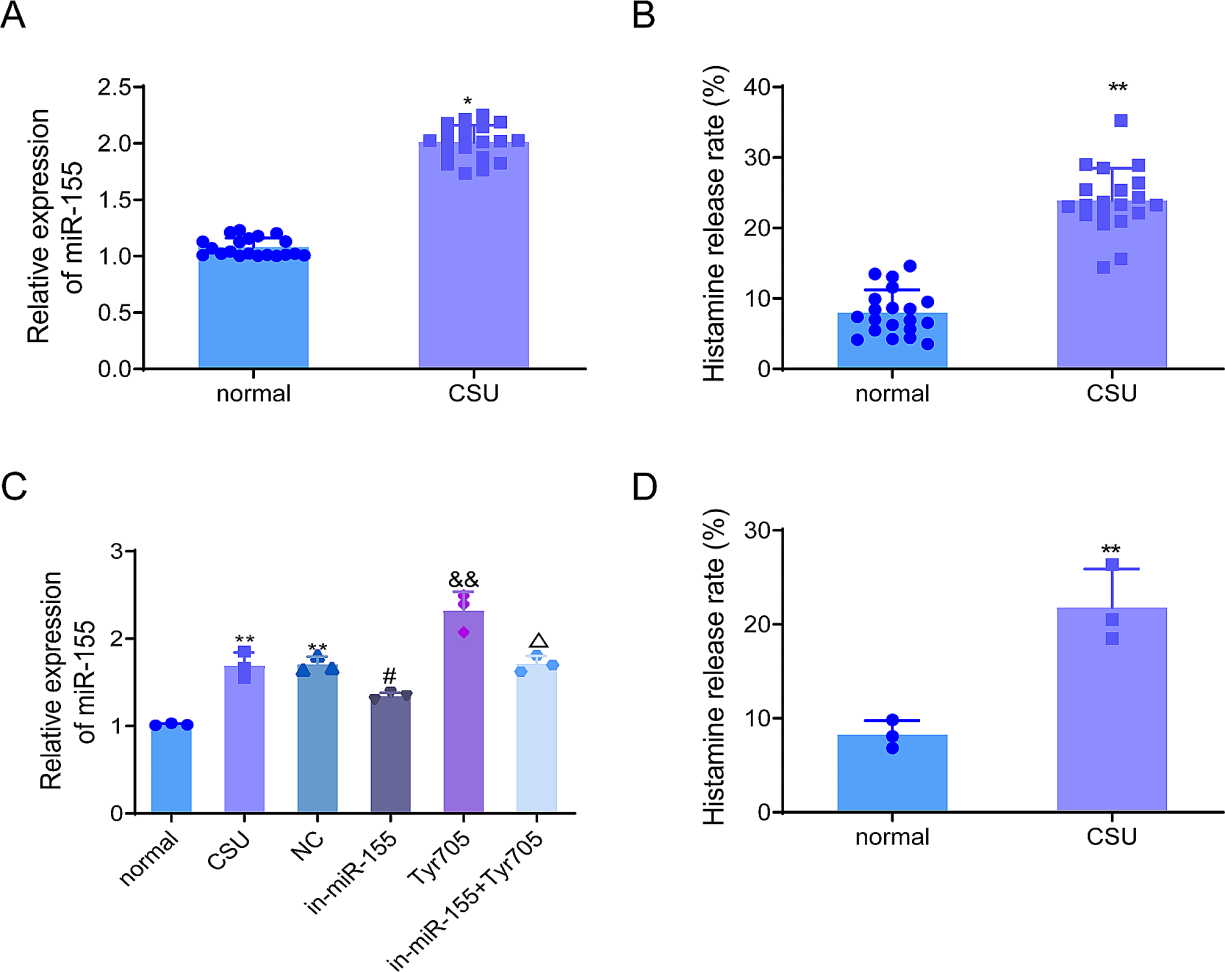
Up-regulated miR-155 expression in skin tissues of patients with CSU and rat models. (**A**) The expression level of miR-155 was detected in skin tissues of patients in the normal and CSU groups was detected by RT-qPCR; (**B**) Histamine release rate in the serum from patients in the normal and CSU groups was measured using histamine release assay; (**C**) The expression level of miR-155 in skin tissues of rats in each group was detected by RT-qPCR; (**D**) Histamine release rate of serum from rats in the CSU group and normal group was detected by means of histamine release assay. *P < 0.05, **P < 0.01 vs. normal group; #*P* < 0.05 vs. NC group, &&*P* < 0.01 vs. CSU group, ∆*P* < 0.05 vs. in-miR-155 group. CSU, chronic spontaneous urticaria

Firstly, RT-qPCR was adopted to assess the expression of miR-155 in the skin tissues of patients with CSU. The results showed that the expression level of miR-155 in the skin tissues of patients with CSU was significantly higher than that of the normal group (*P* < 0.05, Fig. 1A), as well as the histamine release rate from the serum (*P* < 0.01, Fig. 1B). Subsequently, the expression level of miR-155 in the skin tissues of rats was examined. The examination results revealed that the expression level of miR-155 derived from the skin tissue of CSU model rats was significantly increased relative to that of the normal group (*P* < 0.05, Fig. 1C). In addition, the serum histamine release rate of CSU rats was significantly higher than that of the normal group (*P* < 0.01, Fig. 1D). The above results indicated that miR-155 expression was up-regulated in the skin tissues of CSU patients and rats.

To further explore the role of miR-155 in CSU, miR-155 in the skin tissues of rats was knocked down by injecting miR-155 inhibitor. RT-qPCR results showed that the expression level of miR-155 in the in-miR-155 group was significantly lower than that of the NC group (*P* < 0.05, Fig. 1C), which indicated that we successfully knocked down the expression level of miR-155 in the skin tissues of CSU rats. Besides, Tyr705 (JAK/STAT signaling pathway agonist) was injected to observe the effect of JAK/STAT signaling on the expression level of miR-155. In brief, compared with the CSU group, Tyr705 intervention up-regulated the expression level of miR-155 in the skin tissues of rats (*P* < 0.01). Furthermore, the expression level miR-155 in the in-miR-155 + Tyr705 group was significantly up-regulated compared with the in-miR-155 group (*P* < 0.05, **Fig. 1C**). The above results suggested that the activation of the JAK/STAT signaling pathway could up-regulate the expression of miR-155 in the skin tissues of CSU rats.

### Knockdown of miR-155 reduces the number and duration of pruritus in CSU rats

The number and duration of pruritus in rats in each group were recorded (Table 2). Briefly, compared with the normal group, the number of pruritus in the CSU group was significantly increased (*P* < 0.05) and the duration of pruritus was prolonged (*P* < 0.05). Compared with the NC group, the number of pruritus in the in-miR-155 group was significantly decreased (*P* < 0.05) and the duration was shortened (*P* < 0.05). In contrast to the in-miR-155 group, the number of pruritus in the in-miR-155 + Tyr705 group was significantly increased, and the duration was prolonged (*P* < 0.05). The results suggested that knockdown of miR-155 alleviated pruritus symptoms in CSU rats, while activation of JAK/STAT signaling reversed the ameliorative effect of knockdown of miR-155 on pruritus symptoms.

**Table 2:** Number and duration of pruritus in rats in each group in one day

Group	Number of pruritus (Time)	Maximum duration of pruritus (min)
normal	0	0
CSU	54.67 ± 3.09*	27.33 ± 1.25*
NC	55.67 ± 3.40*	26.67 ± 0.47*
in-miR-155	20.00 ± 2.16^*#^	17.67 ± 2.62^*#^
Tyr705	88.00 ± 5.10^*&^	44.33 ± 2.05^*&^
in-miR-155 + Tyr705	57.33 ± 4.11^*∆^	29.67 ± 2.05^*∆^

Note: *P < 0.05 vs. normal group; #*P* < 0.05 vs. NC group, ^&^*P* < 0.05 vs. CSU group, ^∆^*P* < 0.05 vs. in-miR-155 group. CSU, chronic spontaneous urticaria; NC, negative control

### Knockdown of miR-155 attenuates the pathological damage of skin tissue in CSU rats

**Fig. 2:**
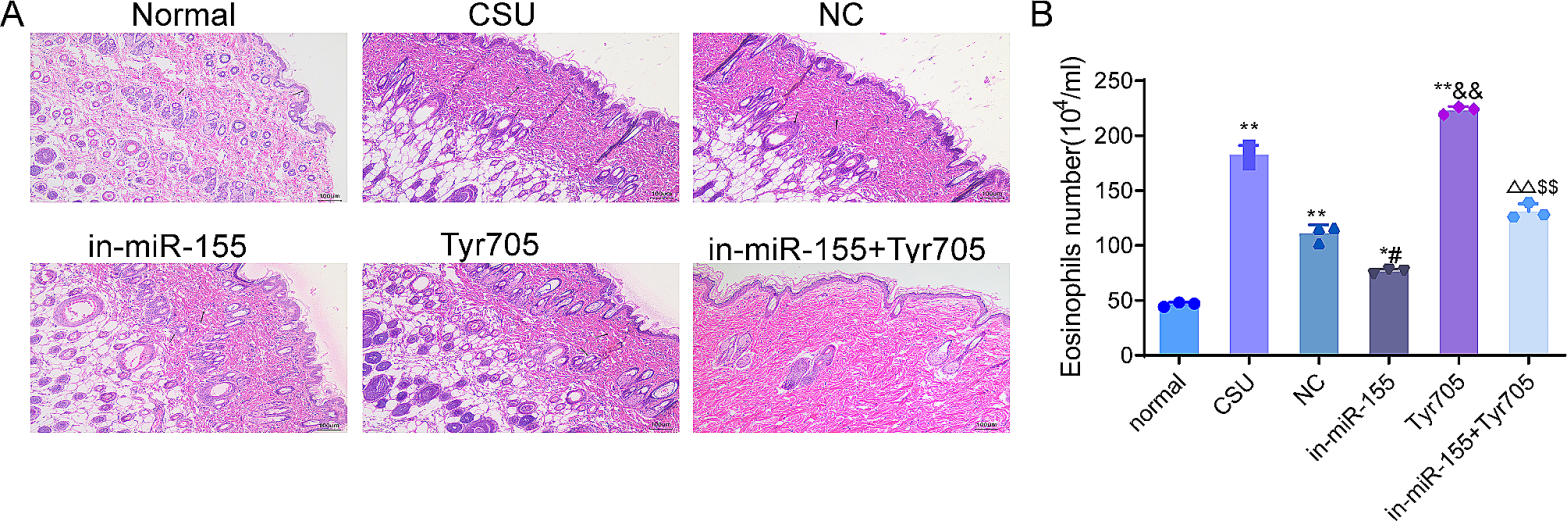
Knockdown of miR-155 attenuates the pathological damage of skin tissues in CSU rats. (**A**–**B**) H&E staining was used to observe the pathological changes of skin tissues in each group of rats and count the number of eosinophils.**P < 0.01, *P < 0.05 vs. normal group; #< 0.05 vs. NC group, ^&&^*P* < 0.05 vs. CSU group, ^∆^*P* < 0.05 vs. in-miR-155 group

H&E staining was applied to observe the skin tissue damage of rats in each group. The results of H&E staining showed that there were no obvious pathological changes, edema, telangiectasis and capillary congestion in the skin tissue of rats in the normal group. In the CSU group and the NC group, dermal edema, telangiectasis and capillary congestion, interstitial inflammatory cell infiltration, increased eosinophils were found in the skin tissues (*P* < 0.01). Compared with the NC group, the pathological damage of skin tissues in the in-miR-155 group was improved, with reduced edema, vascular congestion, and number of eosinophils (*P* < 0.05). This indicated that inhibition of miR-155 expression could reduce skin damage in CSU rats. In addition, compared with the CSU group, Tyr705 group showed aggravated pathological damage, severe dermal edema, telangiectasis, and significantly increased number of eosinophils (*P* < 0.01). In comparison with the Tyr705 group, the pathological damage of the in-miR-155 + Tyr705 group was alleviated to some extent, with a reduction in dermal edema, telangiectasis, and inflammatory cell infiltration (Fig. 2A–B).

### Knockdown of miR-155 reduces the levels of inflammatory factors in serum of CSU rats

**Fig. 3:**
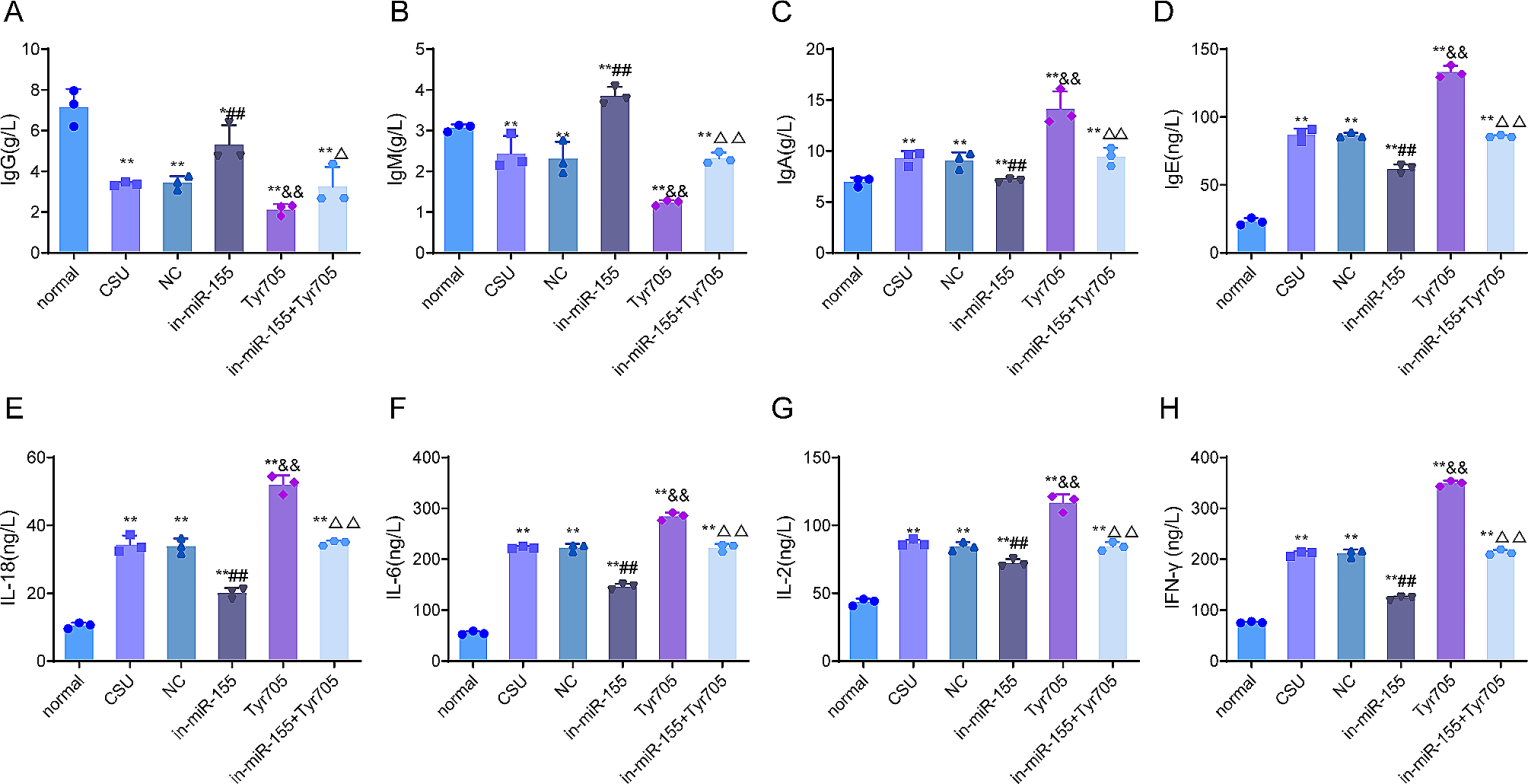
Knockdown of miR-155 reduces the level of inflammatory factors in the serum of CSU rats. (**A**–**C**) The levels of IgG (**A**), IgM (**B**) and IgA (**C**) in the serum of rats in each group were detected by immunoturbidimetric assay; (**D**–**G**) The levels of IgE (**D**), IL-18 (**E**), IL-6 (**F**), IL-2 (**G**) and IFN-γ (**H**) in the serum of rats in each group were detected by ELISA. **P < 0.01 vs. normal group; ##*P* < 0.01 vs. NC group, ^&&^*P* < 0.01 vs. CSU group, ^∆∆^*P* < 0.01 vs. in-miR-155 group. IgG, immunoglobulin G; IgM, immunoglobulin M; IgA, immunoglobulin A; IL, interleukin; IFN-γ, interferon-gamma

Immunoglobulins and inflammatory factors are closely related to the progression of CSU [19]. We investigated the effects of miR-155 on immunoglobulins and inflammatory factors in the serum of CSU rats. Specifically, compared with the normal group, the serum levels of IgG and IgM were decreased and the level of IgA was significantly increased in CSU rats (*P* < 0.01). Compared with the NC group, the levels of IgG and IgM were significantly increased, while the level of IgA was significantly decreased in the in-miR-155 group, (*P* < 0.01). Relative to the CSU group, IgG and IgM levels were significantly lower, whereas IgA levels were significantly higher in the Tyr705 group (*P* < 0.01, Fig. 3A–C). Notably, Tyr705 reversed the increase in IgG and IgM levels and the decrease in IgA levels caused by knockdown of miR-155.

In addition, the serum levels of IL-18, IL-6, IL-2, IFN-γ, and IgE were significantly increased in CSU rats rather than in the normal group (*P* < 0.01), while knockdown of miR-155 reduced the serum levels of IL-18, IL-6, IL-2, IFN-γ and IgE in CSU rats (*P* < 0.01). It was worth noting that Tyr705 intervention reversed the inhibitory effect of knockdown of miR-155 on inflammatory factors (Fig. 3D–H). The above findings suggested that knockdown of miR-155 could reduce the serum levels of inflammatory factors in CSU rats.

### Effect of knockdown of miR-155 on T cell subsets in peripheral blood of CSU rats

**Fig. 4:**
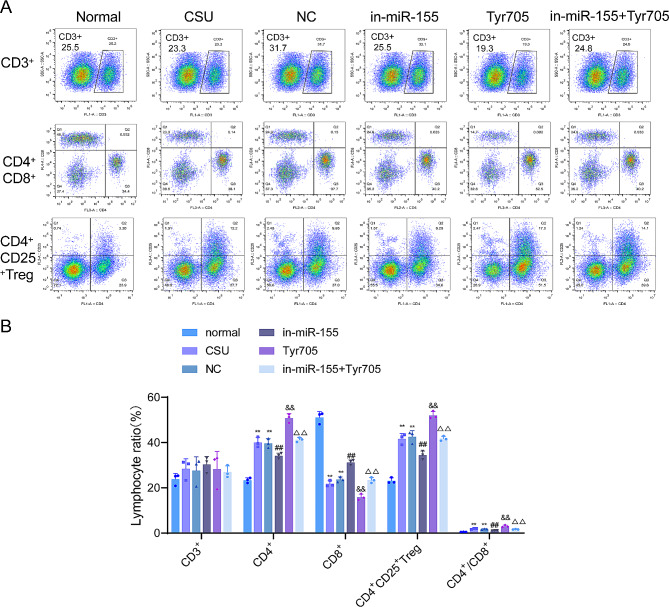
Effect of knockdown of miR-155 on T cell subsets in the serum of CSU rats. (**A**–**B**) The changes in CD3+, CD4+, CD8 + and CD4 + CD25 + T cell subsets in the blood of rats in each group were detected by flow cytometry. **P < 0.01 vs. normal group; ##*P* < 0.01 vs. NC group, ^&&^*P* < 0.01 vs. CSU group, ^∆∆^*P* < 0.01 vs. in-miR-155 group

Flow cytometry was utilized to examine the effect of knocking down miR-155 on T cell subsets in the serum of CSU rats. Briefly, there was no significant difference in CD3 + T cells in the blood samples of rats in each group. Besides, compared with the normal group, the CD4 + and CD4 + CD25 + T cells were increased (*P* < 0.01), the CD8 + T cells were decreased (*P* < 0.01), and the ratio of CD4+/CD8 + was increased (*P* < 0.01) in the peripheral blood of CSU rats. Knockdown of miR-155 decreased CD4 + and CD4 + CD25 + T cells (*P* < 0.01), increased CD8 + T cells (*P* < 0.01), and decreased CD4+/CD8 + ratio (*P* < 0.01) in the peripheral blood of CSU rats. Furthermore, compared with the in-miR-155 group, the CD4 + and CD4 + CD25 + T cells were increased (*P* < 0.01), the CD8 + was decreased (*P* < 0.01), and the CD4+/CD8 + ratio was increased in the in-miR-155 + Tyr705 group (*P* < 0.01, Fig. 4A–B).

### Knockdown of miR-155 inhibits the JAK/STAT signaling pathway in skin tissues of CSU rats

**Fig. 5:**
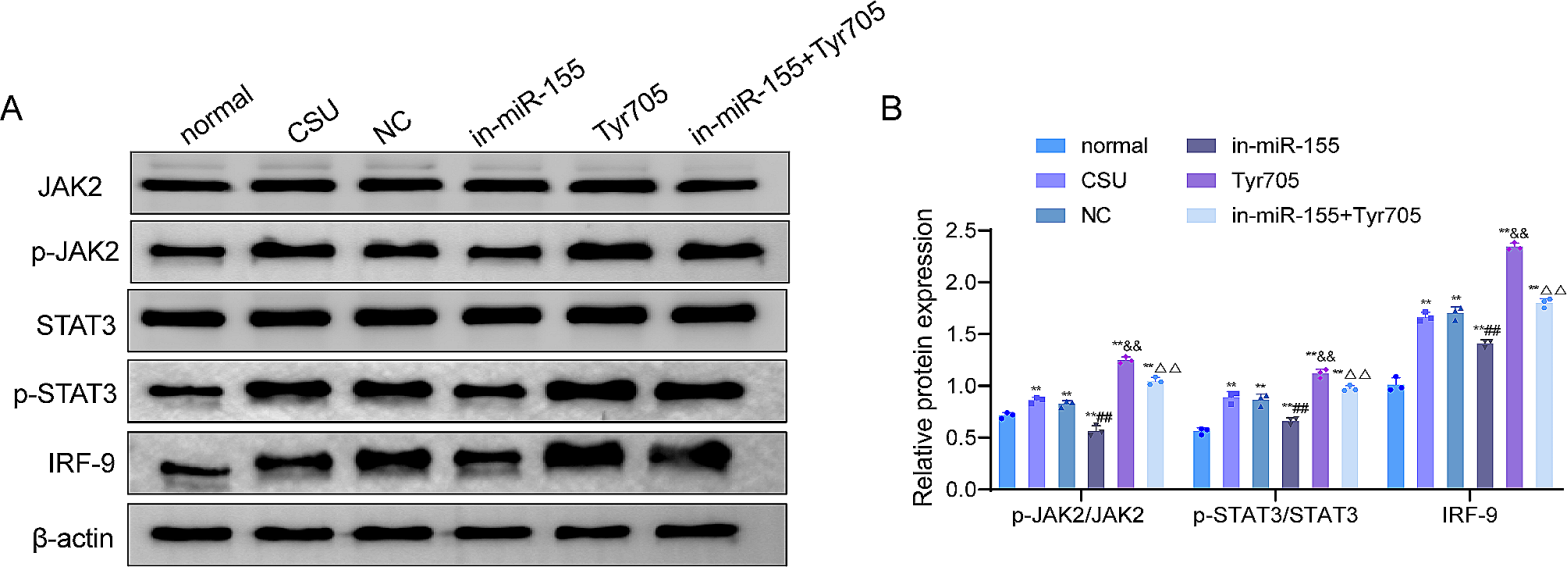
Knockdown of miR-155 inhibits the JAK/STAT signaling pathway in the skin tissues of CSU rats. (**A**–**B**) The protein expression levels of JAK2, p-JAK2, STAT3, p-STAT3, and IRF-9 were detected in the skin tissues of rats in each group by western blot. **P < 0.01 vs. normal group; ##*P* < 0.01 vs. NC group, ^&&^*P* < 0.01 vs. CSU group, ^∆∆^*P* < 0.01 vs. in-miR-155 group. JAK, Janus kinase; STAT, signal transducer and activator of transcription

The previous results suggested that JAK/STAT signaling was closely related to the mechanism of action of miR-155 in CSU. To further reveal the role of JAK/STAT signaling, we examined the expression levels of proteins related to the JAK/STAT signaling pathway in the skin tissues of rats in each group. The examination outcomes displayed that the ratios of p-JAK2/JAK2 and p-STAT3/STAT3 and the expression level of IRF-9 were significantly higher in the skin tissues of CSU rats than those in the normal group (*P* < 0.01). Knockdown of miR-155 decreased the expression of IRF-9 protein and the ratios of p-JAK2/JAK2 and p-STAT3/STAT3 in the skin tissues of CSU rats (*P* < 0.01). Moreover, the expression of IRF-9 protein and the ratios of p-JAK2/JAK2 and p-STAT3/STAT3 were significantly increased in the skin tissues of rats in the Tyr705 group compared with the CSU group (*P* < 0.01). In addition, the in-miR-155 + Tyr705 group of rats exhibited increased expression of IRF-9 protein and up-regulated ratios of p-JAK2/JAK2 and p-STAT3/STAT3 compared with the in-miR-155 group (*P* < 0.01, Fig. 5A and B). Collectively, knockdown of miR-155 inhibited the JAK/STAT signaling pathway in the skin tissues of CSU rats.

## Discussion

Due to symptoms such as severe pruritus and recurrent lesions, CSU seriously affects the quality of life of patients, causing sleep disorders, anxiety, depression, and social dysfunction [20]. The current pharmacological treatments for CSU were mainly achieved by antagonizing histamine, lowering IgE to block mast cell activation, or regulating immune response and inflammatory response [21]. However, some patients still respond poorly to existing pharmacologic treatments [5]. Therefore, it is necessary to explore new therapeutic strategies to treat patients with CSU. In this study, we constructed a rat model of CSU. The number and duration of pruritus of CSU rats were significantly increased compared with the normal group, the rate of histamine release was up-regulated, the skin tissues were pathologically damaged, and eosinophils were increased. This indicated that the CSU model was constructed successfully. Furthermore, we observed that the expression of miR-155 was up-regulated in the skin tissues of CSU patients and rats, suggesting that miR-155 might be involved in the pathological mechanisms of CSU.

Inflammatory response is a major pathological factor in CSU, involving complex interactions among pro-inflammatory mediators, cytokines, and adhesion molecules. These molecules regulate specific processes of cellular infiltration and vasoreactivity, culminating in hypersensitivity reactions [22, 23]. Most CSUs fall into the category of IgE-mediated type I hypersensitivity diseases. The cells involved in type I hypersensitivity reactions mainly include a variety of inflammatory cells such as mast cells, basophils, lymphocytes, eosinophils and neutrophils [24, 25]. Among them, mast cells are mainly located in the skin and submucosal layer. The same antigen entering the organism will specifically bind to the Fab segment of IgE, resulting in the activation of mast cells, the release of a variety of inflammatory mediators such as histamine, leukotrienes, bradykinin, prostaglandins and 5-hydroxytryptophan, and the secretion of a series of cytokines such as IL-2, IL-4, IL-5, IL-6, IL-15, IL-18 and chemokines. Finally, severe inflammatory hypersensitivity reactions are triggered, leading to pruritus, skin mucosal edema and damage [26]. In the present study, the serum histamine release and the levels of inflammatory factors (IL-18, IL-6, IL-2, IFN-γ, and IgE) in CSU rats were significantly elevated, which is consistent with previous reports.

Abnormalities in T-lymphocyte phenotypes are important indicators of immune regulation disorder [27]. CD4 + T cells are involved in cellular immunity and inflammatory responses, and CD8 + T cells can specifically kill infected cells [28]. The balance of CD4+ /CD8 + plays an important role in the progression of CSU. In our study, the CD4 + T cells increased, the CD8 + T cells decreased, and the ratio of CD4+/CD8 + increased in the peripheral blood of CSU rats. These results suggested that immune function of CSU rats was disturbed.

As a member of the non-coding RNA family, miR-155 was reported in early studies to be involved in the inflammatory responses via Toll-like receptor ligands, inflammatory cytokines, and specific antigens [29, 30]. For example, Ryan M et al. [31] proposed that miR-155 promoted the development of inflammatory T cells in rats model of autoimmune encephalomyelitis, enhanced inflammatory responses, and led to disruption in the immune system. Therefore, miR-155 is considered as a potential target for the treatment of autoimmune diseases. Also, Li et al. [32] pointed out that high expression of miR-155 promoted ocular inflammation in Graves’ ophthalmopathy. In this study, miR-155 was significantly over-expressed in skin tissues of CSU patients and rats. Knockdown of miR-155 alleviated the skin damage and inflammatory responses in CSU rats. This suggested that miR-155 promoted autoimmune responses in CSU rats, similar to previous studies.

The JAK/STAT signaling pathway, an important pathway involved in cellular signaling, plays a key role in regulating immune responses, cell proliferation and differentiation [33]. Feng H. et al. indicated that significantly enhanced JAK/STAT signaling pathway activity and STAT3 phosphorylation level in the skin tissues of CSU rats triggered an inflammatory response [16]. A previous study reported that miR-155 could directly bind to STAT3 in autoimmune uveitis mice model, and the expression of mature miR-155 was defective in the absence of STAT3 [34]. In this study, Tyr705 intervention reversed the attenuating effect of knockdown of miR-155 on skin damage and inflammatory response. After inhibiting the expression of miR-155, the levels of JAK2 and STAT3 phosphorylation were reduced and JAK/STAT signaling was suppressed in the skin tissues of CSU rats. The above findings suggested that miR-155 mediated the JAK/STAT signaling pathway involved in the development of CSU.

There are some limitations in this study. Firstly, this study lacked the support of relevant in vitro experiments and failed to fully replicate the complexity of human immune responses and disease mechanisms. Therefore, our findings may not be directly translatable to human patients without further validation. Secondly, we did not inhibit JAK/STAT3 signaling to verify the regulatory effect of miR-155 on JAK/STAT3, so further corresponding experiments are needed to deeply explore and verify the role and mechanism of miR-155 in CSU. Thirdly, the long-term safety, efficacy, and potential off-target effects of sustained miR-155 blocking remain unexplored. Hence, longitudinal studies are necessary to understand the full spectrum of implications associated with miR-155 inhibition over extended periods. Acknowledging these limitations is crucial for interpreting our study’s findings within the appropriate context and underscores the need for further research. Future studies should aim to address these limitations by incorporating long-term analyses, human clinical trials, broader examinations of miR-155’s role in immune regulation, and considerations of genetic and environmental factors influencing CSU.

## Conclusion

To sum up, miR-155 promoted skin damage and inflammatory responses in CSU rats, and its effects might be achieved by activating the JAK/STAT signaling pathway. This study provides a fundamental basis for miR-155 serving as a target for the treatment of CSU.

## Data Availability

The datasets used during the study are available from the corresponding author upon reasonable request.
